# Development and feasibility assessment of a virtual reality-based aerobic exercise program with real-time pulse rate monitoring on hemodynamic and arterial stiffness in healthy people: a pilot study

**DOI:** 10.3389/fdgth.2024.1356837

**Published:** 2024-04-08

**Authors:** Kornanong Yuenyongchaiwat, Natsinee Sermsinsathong, Preeyaphorn Songsorn, Noppawan Charususin, Sasipa Buranapuntalug, Chatchai Buekban, Chusak Thanawattano

**Affiliations:** ^1^ Physiotherapy Department, Faculty of Allied Health Sciences, Thammasat University, Pathumthani, Thailand; ^2^ Thammasat University Research Unit for Physical Therapy in Respiratory and Cardiovascular Systems, Thammasat University, Pathumthani, Thailand; ^3^ Biomedical Electronics and Systems Research Team Assistive Technology and Medical Devices Research Group, National Electronics and Computer Technology Center (NECTEC), Pathumthani, Thailand

**Keywords:** virtual reality, exercise therapy, health promotion, rehabilitation, hemodynamics, pulse wave velocity, dyspnea

## Abstract

**Introduction:**

Virtual reality (VR) exercises are reportedly beneficial as a physical activity tool for health promotion and rehabilitation, and can also help individuals exercise under professional supervision. We developed and investigated the potential feasibility of a VR-based aerobic exercise program using the XBOX ONE console and Kinect sensor with real-time pulse rate monitoring. The VR setting consisted of two-dimensional (2D) environments via computer, laptop, or television screens. In addition, the study investigated the potential feasibility of the VR-based exercise program on hemodynamic response and arterial stiffness in healthy participants of various ages.

**Methods:**

Healthy participants (*n* = 30) aged > 18 years were enrolled in the VR exercise-based program. All participants were required to wear a polar heart rate (HR) monitor set for moderate-intensity exercise, targeting 40%–59% of their HR reserve. Hemodynamic and arterial stiffness (pulse wave velocity) were noninvasively measured. The Borg scale rate of perceived exertion (RPE) was also assessed.

**Results:**

Following a VR-guided exercise routine, all participants performed moderate-intensity exercise with no adverse health outcomes during or after the exercise. The effects of VR-based aerobic exercise extended beyond enhanced central hemodynamic and arterial stiffness. However, neither hemodynamic nor arterial stiffness showed significant differences before and after the VR exercise, except for a higher RPE response following the exercise program.

**Conclusion:**

VR-based aerobic exercise with pulse rate monitoring is a promising physical activity tool to induce physiological changes and impact dyspnea scales and is also feasible for administration to healthy populations.

## Introduction

The World Health Organization (WHO) has recommended that both the general population and individuals with specific health conditions should increase their physical activity through exercise. WHO's reports indicate that a quarter of adults fail to meet the recommended levels of physical activity; it was the third in women and the fourth in men who could not accomplish the recommendation (1). Additionally, people individuals who do not meet the recommended physical activity level reportedly have a 20%–30% increased risk of mortality compared to those who achieve physical activity (1).

To address these challenges and enhance exercise adherence, this study explores the potential of virtual reality (VR) technology. VR enables individuals to immerse themselves in virtual environments and engage with various scenarios. Furthermore, VR has demonstrated the potential to improve the overall exercise experience (2, 3). VR exercise applications offer several advantages, such as real-time feedback and diverse exercise options. These features can help combat motivational challenges and reduce the likelihood of boredom, ultimately enhancing exercise adherence (4, 5). VR programs have applications in healthcare for enhancing physical activity and rehabilitation (6, 7). While VR holds considerable promise in the healthcare sector, its widespread adoption faces challenges due to the high cost of the devices, making them less accessible, especially for individuals requiring supervision from healthcare providers due to health issues.

Exercise plays a role in the prevention of cardiovascular disease by improving endothelial function. Increased nitric oxide and the sheering force of blood flow are mediated through evoked endothelium-dependent vasodilation in blood flow, thereby reducing sympathetic nerve activity (8–10). Collectively, arterial stiffness and the resulting central hemodynamic effects have been linked to adverse cardiovascular events, including coronary artery disease, stroke, and hypertension (11, 12). Pulse wave velocity (PWV) is a non-invasive method used to assess the speed of arterial pressure waves traveling along the aorta and large arteries by measuring arterial stiffness (13, 14) PWV has been widely used as a clinical indicator of cardiovascular risk (15–17), and both carotid-femoral PWV and brachial-ankle PWV (baPWV) are commonly used as predictive techniques. In East Asia, baPWV is widely used in the general population and high-risk populations (18). Consequently, the acute effect of VR-based aerobic exercise on central hemodynamic and peripheral PWV values seems necessary to evaluate the feasibility and possible beneficial or adverse acute effects of exercise. Although the acute effects of VR-based aerobic exercise have received attention, factors contributing to hemodynamic changes and arterial stiffness in different age groups may differ (19, 20). Therefore, the objective of this study was to create and assess the viability of VR-based exercise programs, incorporating real-time heart rate (HR) monitoring within a healthy participant group. We hypothesize that, after performing VR-based aerobic exercise, increased hemodynamic responses and arterial stiffness will be observed in participants.

## Materials and methods

### Participants

This study aimed to develop VR-based aerobic exercise and explore the hemodynamic changes and arterial stiffness in different ages. This pilot study enrolled 30 participants, with 10 participants in each age group: (1) young adults (18–34 years), (2) middle-aged adults (35–59 years), and (3) older adults (60–75 years) (21). Participants with a history of respiratory exacerbation before 6 months, resting HR greater than 120 beats per min (bpm) (22, 23), crisis hypertension (i.e., >180/120 mmHg) (24), those using assistive devices such as a cane or walker, those having uncontrollable blood glucose (i.e., fasting blood sugar greater than 130 mg/dl) (25), a history of cardiopulmonary stroke, or those with psychiatric conditions diagnosed by a physician were excluded. This study protocol was approved by the Human Research Ethics Committee of Thammasat University (Science), COA No. 076/2565. The Thai Clinical Trials Registry is TCTR20230601005. Informed consent was obtained from all participants involved in the study. Written informed consent has been obtained from the participants to publish this paper.

### Development of VR-based aerobic exercise and software development tools

The composition of VR hardware is as follows:

• Mini-PC Android board: Employed for software installation.
• Webcam: A USB camera to capture the participant's images during the exercise.
• Air Mouse G10S (wireless 2.4G) Remote Control.
• Television with HDMI compatibility: Displayed the VR coach and used for on-screen heart rate (HR) monitoring via an HDMI connection.
• Polar Verity Sense (Optical Heart Rate Sensor): A Bluetooth-connected wristband that monitors HR during exercise, with an appropriate range tailored to the user-specific exercise program.

The VR-based aerobic exercise system consisted of an armband HR sensor, web camera, display screen, and Android mini-PC with the required software application. After wearing the HR sensor, the software application guided the user to turn on the device and pair it with the software on the mini-PC via Bluetooth. The software was developed using the Dart programming language (Dart.js) to run on the Android OS. A webcam captured video footage of the user engaged in physical exercise. The video was displayed live on the right side of the display as feedback to allow the user to compare their movement with the guiding video clip playing on the left side of the screen.

**Figure 1:**
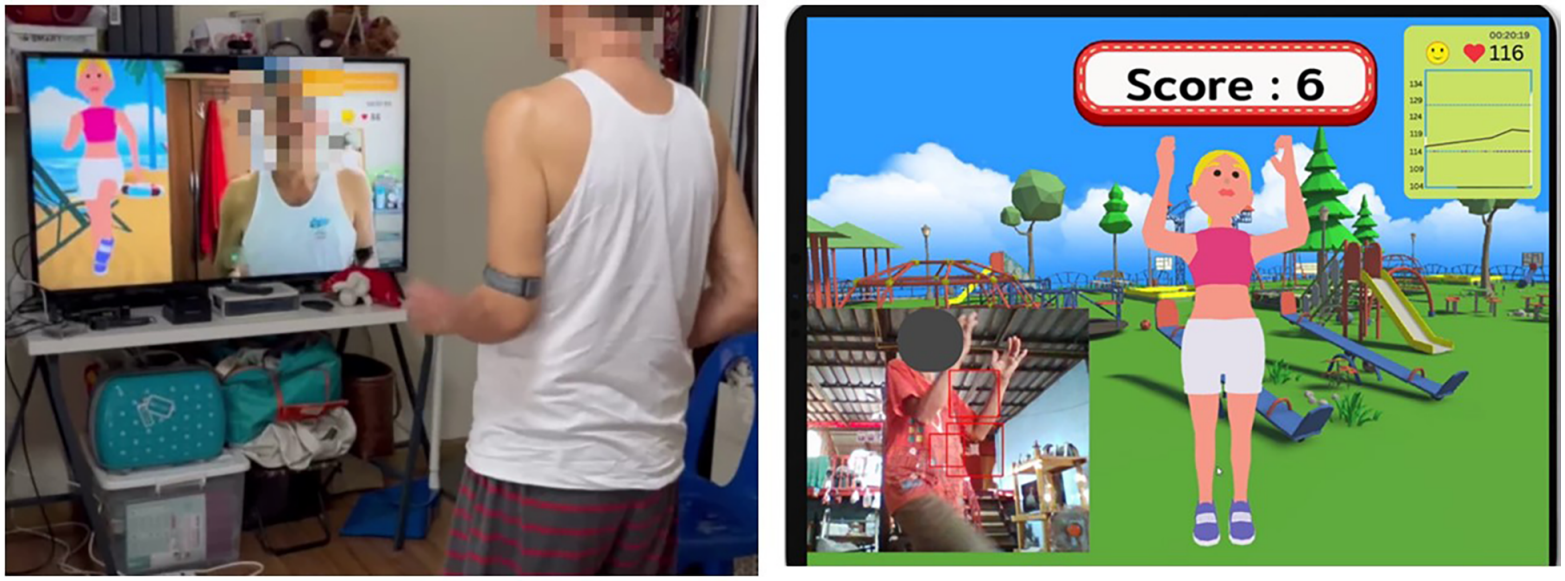
Virtual reality-based aerobic exercise.

The HR monitor was attached to the left upper arm. A real-time HR was displayed on the screen during exercise and the marching intensity was adjusted accordingly using HR monitor reading. The HR data was shown as a transparent time series overlay on the running video. This real-time feedback notified the participant if their HR was too high or too low during the exercise (Figure 1).

The Polar Verity Sense (Polar Electro, Kempele, Finland), functioning as an optical HR monitor armband for VR exercises, has been previously established as reliable. It has shown a high intraclass correlation coefficient and has been deemed valid based on the Concordance Correlation Coefficient (26).

**Figure 2:**
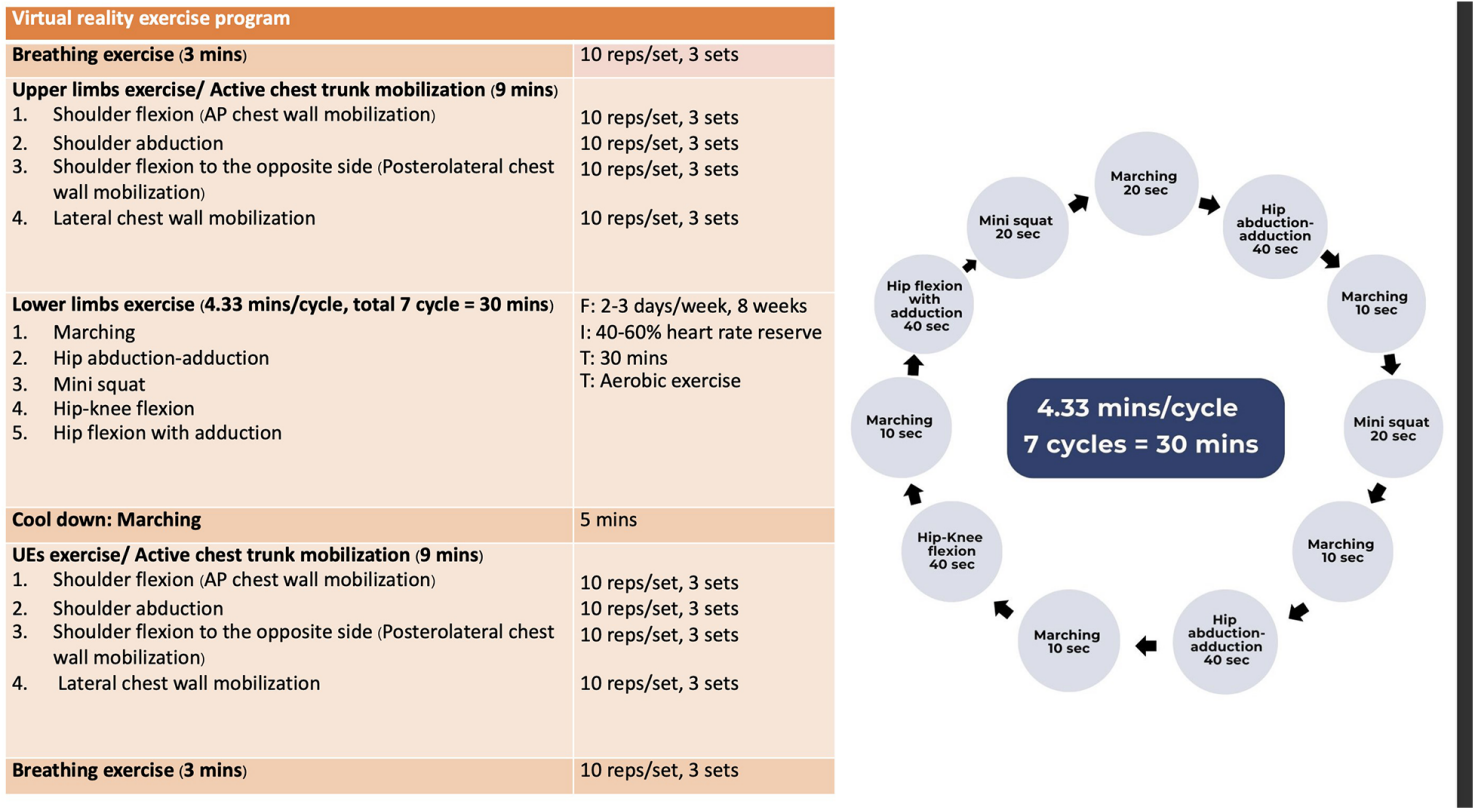
The virtual reality-based aerobic exercise protocols.

The exercise intensity was set at 40%–59% of the HR reserve, representing a definition of moderate aerobic exercise (27). The aerobic exercise program was tailored and refined based on findings from previous research studies (28, 29). Furthermore, the protocol received endorsement and approval from experts in cardiac rehabilitation, including professionals in physical therapy and physical medicine and rehabilitation. The VR-based aerobic exercise program started with a breathing exercise (i.e., slow breathing exercise) for 3-min, followed by a series of warm-up exercises conducted with the upper limbs for 9-min. A 30-min aerobic exercise with moderate intensity exercise is then performed, followed by a cool-down exercise involving marching for 5-min and upper limbs exercise for 9-min. Finally, breathing exercises are performed to conclude the session. Figure 2 illustrates the VR-based aerobic exercise program.

### Procedures

The primary objective of this study was to ascertain the feasibility and safety of VR-based aerobic exercises in healthy participants in different age groups. As part of the assessment, all participants underwent hemodynamic evaluations before and after engaging in VR-based aerobic exercises using PhysioFlow® Enduro TM and VP-1000 Plus. The hemodynamic parameters were assessed by a portable Physioflow® Enduro TM (Manatec Biomedical, PhysioFlow Enduro, Paris, France) which is a non-invasive method employed to detect changes in cardiac output (CO), stroke volume (SV), and HR. It relies on transthoracic bioimpedance as the primary measure of cardiovascular responses. To conduct these measurements, participants had sensors attached to their chest wall and were instructed to sit quietly in a chair for 10-min. Subsequently, CO, SV, and HR were recorded.

Arterial wall stiffness was assessed using the VP-1000 Plus (Omron Healthcare, Japan), and brachial-ankle pulse wave velocity (baPWV) was measured as part of vascular screening. It is worth noting that baPWV is closely linked to carotid-femoral pulse wave velocity (cf PWV) (30) and can serve as a risk indicator for cardiovascular disease (31). To measure baPWV, pressure cuffs on both arms and ankles measure pulse waves of the brachial and posterior tibial arteries while participants were instructed to lie on their backs for a 5-min period. The present study included the right baPWV measurement for analysis,

After switching on the VR hardware, all participants were required to connect the HR monitor above the left elbow. Resting HR was shown on the screen and then the instructor set 40%–59% of heart rate reserve (HRR) for moderate-intensity exercise. This study used an equation (220—age) to determine the age-predicted maximal heart rate (32). The VR exercise protocol proposed aerobic activities started with breathing exercises, then participants followed the VR exercise protocol for 60-min.

Following the 60-min session of VR-based aerobic exercise, participants were allowed a 10-min rest period, after which they were invited to undergo evaluations of hemodynamic changes and vascular stiffness using PhysioFlow® Enduro TM and VP-1000 Plus, respectively. Additionally, the Borg rate of perceived exertion (RPE) was employed to assess dyspnea levels both before and after exercise.

### Statistical analysis

The normal distribution of data was assessed using the Shapiro-Wilk test, and a two-way repeated mixed ANOVA was conducted to compare within and between groups (2 × 3). The statistical analysis was performed using IBM SPSS Statistics for Windows version 24.0 software, with *p* < 0.05 considered as statistically significant.

## Results

The three participant groups are detailed in Table 1. The study included 30 healthy participants, with 10 participants in each group. Out of the 30 participants, 20 were female and the average age was 44 years (range 20–75 years). All participants achieved the VR-based aerobic exercise protocol by maintaining a 40%–59% HRR without any adverse outcomes. All participants achieved their expected exercise intensity (40% and 59% of their HRR for moderate intensity exercise; 129–150 bpm for young adults, 113–133 bpm for middle adults, and 104–120 bpm for older adults. During VR-based aerobic exercise, the minimal and maximal HR achieved was 115–143 bpm for young adults, 99–126 bpm for middle adults, and 98–113 for older adults. There were no significant differences in sex and body mass index among three groups of age (i.e., young adults, middle adults, and older adults) in the present study (*p* = .058 and *p* = .521, respectively).

**Table 1:** Characteristics data of the study.

	Total (*N* = 30) Mean ± SD	Young adults (*n* = 10) Mean ± SD	Middle adults (*n* = 10) Mean ± SD	Older adults (*n* = 10) Mean ± SD
Age (years)	44.0 ± 19	23 ± 2	43 ± 7	66 ± 5
BMI (kg/m^2^)	24.8 ± 6.0	23.1 ± 4.6	26.2 ± 8.4	25.2 ± 4.3
Sex				
Female (%)	20 (66.7)	4 (20.0)	9 (45.0)	7 (35.0)
Male (%)	10 (33.3)	6 (60.0)	1 (10.0)	3 (30.0)

BMI, body mass index; SD, standard deviation.

The PhysioFlow® device failed to measure CO in seven participants, likely due to poor signal quality or electrode contact failure (three in the middle-aged group and four in the older age group). Accordingly, the SV and CO were analyzed in the remaining 23 participants. The results revealed significant increases in SBP and DBP after performing VR-based exercise program in young adulthood (*Δ*6 mmHg and *Δ*3 mmHg, respectively), and high HR values were noted in middle-aged adults (*Δ*9 bpm). In addition, significant variations were observed in dyspnea scores post-VR exercise, with RPE scores of 6–12 (*p* < .001), as shown in Table 2.

**Table 2:** Comparison of hemodynamic responses and pulse wave velocity between subgroups of cases based on different aged groups.

	Young adults		*p*-value	Middle adults		*p*-value	Older adults		*p*-value	*p*-value^a^	*p*-value^b^	*p*-value^c^
	Pre-test mean ± SD	Post-test mean ± SD		Pre-test mean ± SD	Post-test mean ± SD		Pre-test mean ± SD	Post-test mean ± SD				
SBP (mmHg)	117 ± 23	123 ± 21	.041	116 ± 17	114 ± 11	.621	124 ± 14	123 ± 15	.724	.217	.957	.237
DBP (mmHg)	68 ± 11	72 ± 10	.047	68 ± 7	66 ± 4	.321	72 ± 9	73 ± 9	.446	.126	.686	.057
HR (bpm)	84 ± 8	86 ± 9	.471	70 ± 10	78 ± 6	.033	71 ± 16	74 ± 134	.601	.073	.006	.263
MAP (mmHg)	87 ± 14	90 ± 13	.116	84 ± 9	83 ± 5	.671	90 ± 13	91 ± 14	.777	.154	.970	.144
PP (mmHg)	49 ± 14	51 ± 11	.542	48 ± 12	48 ± 10	.878	52 ± 9	50 ± 8	.192	.639	.858	.771
baPWV (cm/s)	1,122 ± 168	1,112 ± 191	.803	1,181 ± 176	1,202 ± 144	.623	1,461 ± 194	1,506 ± 171	.287	.248	<.001	<.001
CO (L/min)	5.8 ± 2.2	6.1 ± 1.7	.051	7.5 ± 3.5	6.0 ± 2.7	.970	5.2 ± 1.8	5.1 ± 1.0	.904	.223	.035	.327
SV (ml)	60.2 ± 25.4	67.9 ± 18.0	.278	82.6 ± 22.5	73.4 ± 11.8	.275	70.9 ± 21.5	63.9 ± 9.3	.442	.443	.604	.251
RPE	6.3 ± 0.5	11.4 ± 1.7	<.001	6.7 ± 1.0	12.9 ± 2.1	<.001	6.6 ± 0.5	12.4 ± 1.3	<.001	.064	.210	.526

SD, standard deviation; SBP, systolic blood pressure; DBP, diastolic blood pressure; HR, heart rate; MAP, mean arterial pressure; PP, pulse pressure; baPWV, brachial-ankle pulse wave velocity; CO, cardiac output; SV, stroke volume; RPE, rate perceived exertion; mmHg, millimetres of mercury; bpm, beat per minute; L/min, Liter per minute; cm/s, centimeters per second; ml, millilitre.

^a^ Compared between young adults and middle adults.

^b^ Compared between young adults and older adults.

^c^ Compared between middle adults and older adults.

Focusing on the age groups, significant differences in baPWV values were exhibited in older adults compared to middle-aged adults and young adults (*Δ*394 and *Δ*304 cm/s, respectively). Additionally, it was found that the young adults had high CO values compared to older adults (*Δ*2.0 L/min).

## Discussion

This study aimed to develop and assess the feasibility of VR-based aerobic exercise programs, incorporating real-time HR monitoring within a healthy participant group. We hypothesized that all participants could achieve their expected moderate intensity during VR-based aerobic exercise and that, there would be increased hemodynamic responses and arterial stiffness observed in healthy populations after performing the VR-based aerobic exercise.

Immersive VR is kinesthetic engagement in performing an exercise that can be immersion in the virtual world (33). The prototype of a VR-based exercise device in the present study allows a user to interact with virtual environments and it offers a low-cost VR. In addition, the development of VR-based aerobic exercises has been successfully studied in healthy participants, and no adverse events were observed in this study. Initially, all participants presented increased HR, SBP, DBP, MAP, PP, PWV, and RPE while practicing the VR-based aerobic exercise program. Additionally, all the participants achieved the target HR through moderate-intensity training provided through the VR program. Regarding the safety of a device that affects hemodynamic changes during exercise, none of the measured parameters, such as SBP, DBP, HR, CO, SV, baPWV values, or RPE, exceeded the exercise termination criteria established by the American College of Sports Medicine (ACSM) (27). The present study found that these parameters showed a modest increase 10-min after exercise. A systematic review and meta-analysis reported that exercise acutely elevates PWV, which peaks immediately after exercise and subsequently diminishes 30-min after exercise (34). In the present study, the mean values pre-exercise ranged from 1,122–1,461 cm/s, and the mean values post-exercise ranged from 1,112–1,506 cm/s. The normal values of baPWV ranged from 1,200–1,630 cm/s in individuals aged between 15 and 88 years old (35). Therefore, the increased baPWV after performing VR based aerobic exercise program is not higher than the normal range of baPWV range. Additionally, we observed a slightly elevated SBP, DBP, and HR after performing a VR-based exercise program for 10-min. Notably, these averages in SBP and DBP are considered normal by the American Heart Association (36). Regarding the subjective assessment, dyspnea scores were assessed before the exercise program and ranged from no exertion at all to extremely light (RPE scale = 6–7). These scores were reevaluated after exercise and ranged from light to somewhat hard (RPE scale = 11–13) (37). Alternatively, the ratings of perceived exertion were significantly greater during VR-based aerobic exercise in healthy participants. Therefore, the perceived exertion or breathlessness experienced during VR exercise, which is a subjective measure, could significantly influence individual responses to the VR exercise, and this may be a sufficient tool for healthy people to enhance cardiovascular responses to exercise.

For the effects of VR exercise on health outcomes, a recent systematic review and meta-analysis of 15 studies indicated a positive effect of VR on physiological (e.g., increases in balance, performance) and psychological (e.g., decreased depression, fatigue) health, and no study reported the negative effects on health in VR exercise (38). Furthermore, VR exercise improves cardiovascular performances such as HR, and blood pressure in patients with heart disease, and patients with respiratory problems (39, 40). Therefore, these findings suggest that VR-based aerobic exercise can help improve cardiovascular response and safety. Consequently, VR-based aerobic exercise might be optimal for enhancing hemodynamic responses to exercise in healthy participants, irrespective of their age group.

Despite the significant insights uncovered, this study also had some limitations. This study primarily focused on the immediate hemodynamic and perceived exertion responses to VR-based aerobic exercise. Long-term effects on health outcomes or safety were not assessed. It is important to conduct follow-up studies to determine if the positive effects observed in this study are sustained over time and if any potential adverse effects emerge with prolonged use. In addition, different sports recommend different heart rate monitors. The Polar Verity Sense Optical Heart Rate Sensor is recommended for swimming; therefore, the Polar H10 Heart Rate Sensor might be beneficial for overall activities (41). Additionally, we did not calculate the sample size, and only healthy participants were recruited because this study aimed to develop and explore the feasibility of a VR device. Therefore, further studies are needed with a sufficient sample size and the clinical application of VR-based aerobic exercise in various populations, including those with heart disease or those who have undergone cardiac surgery.

## Conclusions

VR-based aerobic exercise in healthy participants was associated with high acceptance and no adverse events. Future research endeavors must encompass a broader scope, encompassing diverse patient demographics, protracted observation periods, and comparative analyses, to comprehensively elucidate the therapeutic potential and safety parameters associated with this innovative exercise modality.

## Data Availability

The original contributions presented in the study are included in the article/Supplementary Material, further inquiries can be directed to the corresponding author.
